# 11例伴t(14;19)(q32;q13)的慢性淋巴细胞白血病患者临床特征

**DOI:** 10.3760/cma.j.issn.0253-2727.2023.05.011

**Published:** 2023-05

**Authors:** 成华 崔, 亚楠 常, 吉 周, 承文 李, 慧君 王, 琦 孙, 玉娇 贾, 庆华 李, 婷玉 王, 录贵 邱, 树华 易

**Affiliations:** 中国医学科学院血液病医院（中国医学科学院血液学研究所），实验血液学国家重点实验室，国家血液系统疾病临床医学研究中心，细胞生态海河实验室，天津 300020 State Key Laboratory of Experimental Hematology, National Clinical Research Center for Blood Diseases, Haihe Laboratory of Cell Ecosystem, Institute of Hematology & Blood Diseases Hospital, Chinese Academy of Medical Sciences & Peking Union Medical College, Tianjin 300020, China

**Keywords:** 白血病，淋巴细胞，慢性, t（14;19）（q32;q13）, IGH::BCL3, 预后, Leukemia, lymphocytic, chronic, t（14;19）（q32;q13）, IGH::BCL3, Prognosis

## Abstract

**目的:**

分析伴t（14;19）（q32;q13）的慢性淋巴细胞白血病（CLL）患者的临床特征。

**方法:**

回顾性分析2018年1月1日至2022年7月30日中国医学科学院血液病医院染色体核型分析结果中伴有t（14;19）（q32;q13）的11例CLL患者的病例资料。

**结果:**

11例患者中，t（14;19）（q32;q13）均导致IGH::BCL3基因重排，多伴有+12或复杂核型，免疫表型积分4～5分者7例，3分者4例。突变频率较高的基因为NOTCH1（3/7）、FBXW7（3/7）和KMT2D（2/7）。极高危组1例，高危组1例，中危组6例，低危组3例。2例患者死亡，7例患者存活，2例患者失访，4例患者治疗期间出现疾病进展或复发。中位首次治疗时间为1个月。

**结论:**

t（14;19）（q32;q13）可涉及IGH::BCL3基因重排，是CLL中少见的重现性染色体异常，可能提示预后不良。

## Abstract (en)

**Objective:**

To analyze the clinicopathological characteristics of 11 cases of chronic lymphocytic leukemia（CLL）with t（14;19）（q32;q13）.

**Methods:**

The case data of 11 patients with CLL with t（14;19）（q32;q13）in the chromosome karyotype analysis results of the Blood Diseases Hospital, Chinese Academy of Medical Sciences from January 1, 2018, to July 30, 2022, were retrospectively analyzed.

**Results:**

In all 11 patients, t（14;19）（q32;q13）involved IGH::BCL3 gene rearrangement, and most of them were accompanied by +12 or complex karyotype. An immunophenotypic score of 4–5 was found in 7 patients and 3 in 4 cases. We demonstrated that CLLs with t（14;19）（q32;q13）had a mutational pattern with recurrent mutations in NOTCH1（3/7）, FBXW7（3/7）, and KMT2D（2/7）. The very-high-risk, high-risk, intermediate-risk, and low-risk groups consisted of 1, 1, 6, and 3 cases, respectively. Two patients died, 8 survived, and 2 were lost in follow-up. Four patients had disease progression or relapse during treatment. The median time to the first therapy was 1 month.

**Conclusion:**

t（14;19）（q32;q13）, involving IGH::BCL3 gene rearrangement, is a rare recurrent cytogenetic abnormality in CLL, which is associated with a poor prognosis.

t（14;19）（q32;q13）是一种重现性染色体异常，可导致IGH::BCL3基因重排，主要见于慢性淋巴细胞白血病（CLL）及其他B细胞恶性肿瘤[1]。CLL患者的预后呈高度异质性，细胞遗传学特征是重要的预后因素[2]。CpG寡脱氧核苷酸（CpG-ODN）联合白细胞介素-2（IL-2）应用于CLL患者的骨髓或外周血细胞培养使核型分析的成功率及染色体异常的检出率大大提高，其中IGH易位受到越来越多的重视[3]–[4]。在CLL中，IGH常见的伙伴基因包括MYC、BCL2、BCL3等。伴t（14;19）（q32;q13）的CLL患者的临床特征和临床意义尚不明确，目前多为小队列报道或病例报道[3]–[7]。本文纳入中国医学科学院血液病医院自2018年至2022年收治的11例伴t（14;19）（q32;q13）的CLL患者，对其临床特征进行归纳总结。

## 病例与方法

1. 病例：检索中国医学科学院血液病医院（中国医学科学院血液学研究所）细胞分子遗传实验室的数据库，收集2018年1月1日至2022年7月30日期间染色体核型分析结果中包含t（14;19）（q32;q13）的CLL病例，并对其染色体核型分析、荧光原位杂交（FISH）、免疫表型、病理组织化学、二代测序结果及临床病例资料进行回顾性分析，对患者进行随访，分析其疗效及首次治疗时间（TTT）等。

2. 免疫表型：免疫表型分析采用多色流式细胞术，应用荧光标记的单克隆抗体孵育单个核细胞，通过流式细胞仪对抗体进行检测。检测的抗体包括CD5、CD10、CD19、CD20、CD22、CD23、CD43、CD200、CD79b、FMC7、sIgM、sIgD、Kappa、Lambda等。

3. 细胞分子遗传学：

（1）染色体核型分析：取患者骨髓（肝素抗凝）或外周血标本3 ml，以（1～3）×10^6^/ml的细胞密度接种于含20％小牛血清（美国Gibco公司产品）的RPMI 1640培养基中，加入CpG-ODN及IL-2[8]，培养72 h，于收获前1 h加入秋水仙酰胺（美国Sigma公司产品），终浓度为0.2 µg/ml，将细胞阻滞在有丝分裂中期。常规收获后制片，胰蛋白酶消化后，用吉姆萨染液染色，完成G显带。通过Ikaros系统（德国Metasystems公司产品）全自动扫描并进行分析，每个标本分析20个中期分裂象，核型描述依据《人类细胞遗传学国际命名体制（ISCN2016）》。复杂核型是指存在≥3种染色体异常，高复杂核型是指存在≥5种染色体异常[9]。

（2）有丝分裂间期FISH：IGH::BCL3双色双融合探针选用英国Cytocell公司产品。常规收获未培养的骨髓或外周血细胞，依据试剂厂商提供的操作说明书完成杂交。由两位医师使用荧光显微镜（Olympus BX51）分别计数100个细胞，对结果进行分析。

（3）有丝分裂中期FISH：探针同有丝分裂间期，采用培养后的骨髓细胞进行染色体核型分析后，标记目标分裂中期的位置，选用IGH::BCL3探针进行杂交。使用荧光显微镜观察并分析分裂中期的探针信号特征。

4. 分子生物学：取患者骨髓或外周血标本提取基因组DNA，采用Sanger测序对IGHV突变情况进行分析。

5. 二代测序：取患者骨髓标本3 ml，提取基因组DNA，利用Ion Torrent测序平台完成高通量测序。测序结果与人类参考基因组Hg19进行比对。

6. 随访：通过查阅病例资料及电话对患者进行随访，随访截止时间为2022年9月1日，2例患者失访。TTT定义为自诊断至首次治疗或末次随访的间隔时间。

7. 统计学处理：统计学分析利用SPSS 23.0软件进行，定量资料用中位数（范围）表示，计数资料用例数表示。

## 结果

1. 临床特征：本研究11例患者中，女5例，男6例，中位年龄55（51～72）岁，仅1例患者大于65岁。4例患者有B症状，9例有淋巴结肿大，7例有脾肿大或肝肿大。3例患者HGB小于100 g/L，所有患者的PLT均>100×10^9^/L。6例患者β_2_-微球蛋白（β_2_-MG）>3.5 mg/L，1例患者TP53基因缺失，1例患者TP53基因突变，4例患者无IGHV基因突变。根据《中国慢性淋巴细胞白血病/小淋巴细胞淋巴瘤的诊断与治疗指南（2022年版）》[2]中的临床分期系统，10例患者临床分期为Rai Ⅰ～Ⅳ期或Binet B～C期。综合分析上述临床病例资料，根据CLL国际预后指数（CLL-IPI）评分系统，低危组3例，中危组6例，高危组1例，极高危组1例。11例患者的具体临床特征见表1。

**表1:** 11例伴t（14;19）（q32;q13）的慢性淋巴细胞白血病患者的临床特征

例号	性别	年龄（岁）	B症状	Rai分期	Binet分期	β_2_-MG	TP53异常（缺失或突变）	IGHV基因突变状态	IGHV使用片段	CLL-IPI评分	危险度分层
1	男	53	盗汗、乏力	Ⅰ	A	1.73	阴性	未突变	V4-39	3	中危
2	男	51	乏力	Ⅱ	B	2.71	缺失，突变	未突变	V4-34	7	极高危
3	男	53	体重减轻	Ⅰ	B	4.86	阴性	未突变	V3-48	5	中危
4	女	72	无	Ⅱ	B	4.99	阴性	未突变	V4-34	6	高危
5	男	61	无	0	A	3.13	阴性	突变	V3-73	0	低危
6	女	54	乏力	Ⅱ	B	2.99	阴性	NA	NA	1	低危
7	女	55	无	Ⅰ	A	4.34	阴性	突变	V4-39	3	中危
8	男	53	无	Ⅱ	B	3.09	阴性	NA	NA	1	低危
9	女	56	无	Ⅲ	C	3.58	阴性	突变	V4-34	3	中危
10	男	57	无	Ⅲ	C	7.86	阴性	突变	V4-39	3	中危
11	女	61	无	Ⅳ	C	4.34	阴性	突变	V4-39	3	中危

**注** B症状：不明原因的发热（体温38 °C以上）、盗汗、体重减轻（6个月内不明原因体重减轻10%以上）；β_2_-MG：β_2_-微球蛋白；CLL-IPI：慢性淋巴细胞白血病国际预后指数；NA：未检测

2. 免疫表型：11例患者均应用流式细胞术进行了免疫表型检测。根据CLL免疫表型积分系统[2],[10]，免疫表型积分4～5分7例，3分4例。4例患者CD5表达减弱，2例患者部分表达CD5，1例患者CD5表达缺失。3例患者部分表达FMC7，2例患者FMC7表达减弱。6例患者表达CD22，3例患者部分表达CD22。4例患者表达CD79b，1例患者部分表达CD79b。11例患者均可见CD23表达，部分患者CD23呈部分表达或表达减弱。11例患者表面免疫球蛋白（sIg）均不表达或表达减弱（表2）。

**表2:** 11例伴t（14;19）（q32;q13）慢性淋巴细胞白血病患者的免疫表型积分

例号	CD5	CD23	FMC7	sIg	CD22/CD79b	积分
1	+	±	±	−	+/+	3
2	dim	+	−	dim	+/+	4
3	dim	+	−	dim	+/−	5
4	+	±	−	−	+/dim	5
5	±	+	dim	dim	+/NA	3
6	+	+	−	dim	±/dim	5
7	+	+	±	−	dim/dim	4
8	dim	dim	±	−	dim/dim	4
9	−	±	−	−	+/+	3
10	dim	dim	−	−	±/+	4
11	±	dim	dim	dim	±/±	3

**注** +：表达；±：部分表达；−：不表达；dim：表达减弱；NA：未做相关检测

3. 细胞遗传学及分子遗传学：所有患者均进行了染色体核型分析及ATM基因、TP53基因、RB-1基因、CEP12、IGH::CCND1等FISH探针的检测。其中3例患者为单纯性t（14;19）（q32;q13），3例患者伴+12，4例患者为复杂核型（图1），仅1例患者伴TP53基因缺失，11例患者均未见RB-1基因、ATM基因的缺失。IGH::CCND1探针的FISH融合信号均为阴性，但核型分析结果提示IGH基因分离。利用IGH::BCL3探针在细胞间期或中期分裂象进行FISH检测，均为阳性，例8的染色体核型及中期、间期FISH结果见图2。

**图1:**
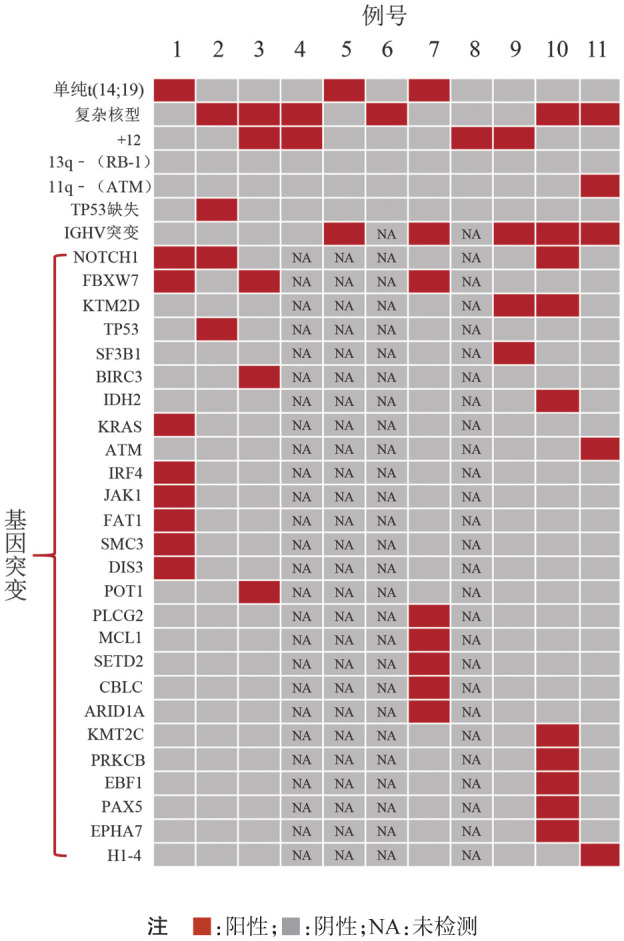
11例伴t（14;19）（q32;q13）慢性淋巴细胞白血病患者的细胞遗传学及分子遗传学结果

**图2:**
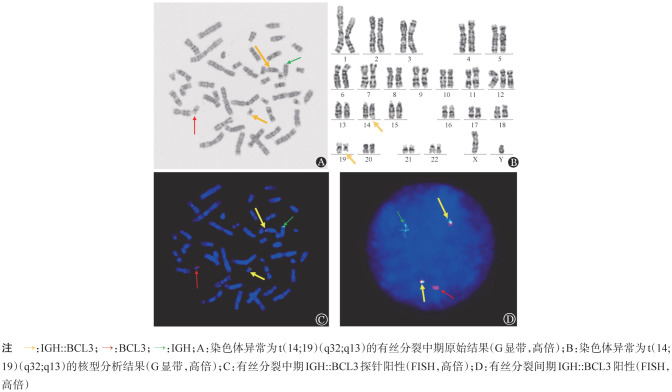
例8的染色体核型及中期、间期FISH结果

4. 分子生物学及二代测序：9例患者进行了IGHV基因突变的检测，4例患者IGHV基因未突变，IGHV基因使用片段为V4-39和V4-34的患者均为4例。7例患者进行了包含80个淋巴瘤相关靶基因的二代测序检测，其中3例患者显示NOTCH1基因突变，3例患者显示FBXW7基因突变，2例患者显示KMT2D基因突变，例2有TP53基因突变（图1）。

5. 治疗与转归：中位随访18（1～55）个月。11例患者中2例失访，2例死亡，7例存活，未失访患者均达到治疗指征并接受了治疗，中位TTT为1（0～19）个月。4例患者在治疗期间出现复发或进展，1例患者接受系统治疗后获得了部分缓解（PR）。化疗方案包括R-CHOP（利妥昔单抗+环磷酰胺+脂质体阿霉素+长春地辛+地塞米松）、FCR（利妥昔单抗+氟达拉滨+环磷酰胺）等，维持治疗药物包括泽布替尼和伊布替尼等（表3）。2例患者参加过临床试验，其中例2因疾病进展参加ABT-199（Venetoclax）的临床试验，23个月后疾病再次出现进展，退出试验。例8参加“BT-1053在复发/难治B细胞淋巴瘤患者中的耐受性与药代动力学Ⅰ期临床研究”，病情相对平稳。

**表3:** 11例伴t（14;19）（q32;q13）慢性淋巴细胞白血病患者的治疗方案及疗效

例号	治疗方案	疗效
1	R-CDOP方案6个疗程；CDOP方案1个疗程；R-CDOP方案1个疗程；FCR方案3个疗程；泽布替尼	复发
2	FCR方案2个疗程；伊布替尼；ABT-199（Venetoclax）临床试验；R-BL方案6个疗程	死亡
3	FCR方案3个疗程	PR
4	伊布替尼	死亡
5	失访	失访
6	失访	失访
7	FCR方案1个疗程；伊布替尼；R-CDOP方案7个疗程；MA方案1个疗程；R-ESHAP方案1个疗程	进展
8	沙利度胺、苯丁酸氮芥；BT-1053Ⅰ期临床试验	SD
9	R-CDOP方案3个疗程；R-CD方案1个疗程	SD
10	FCR方案6个疗程；泽布替尼	进展
11	泽布替尼	进展

**注** R-CDOP：利妥昔单抗+环磷酰胺+脂质体阿霉素+长春地辛+地塞米松；CDOP：环磷酰胺+脂质体阿霉素+长春地辛+地塞米松；FCR：利妥昔单抗+氟达拉滨+环磷酰胺；R-BL：利妥昔单抗+苯达莫司汀+来那度胺；MA：甲氨蝶呤+阿糖胞苷；R-ESHAP：利妥昔单抗+依托泊苷+顺铂+甲泼尼龙+阿糖胞苷；R-CD：利妥昔单抗+环磷酰胺+脂质体阿霉素；PR：部分缓解；SD：疾病稳定

## 讨论

t（14;19）（q32;q13）是B细胞恶性肿瘤中的重现性染色体异常[1]。目前，关于该染色体异常在CLL及其他B细胞增殖性疾病中的研究仅有为数不多的小队列报道或病例报道。研究提示，t（14;19）（q32;q13）（IGH::BCL3）阳性的CLL可能是一个独特的亚组，与典型的CLL有不同的特征，如患者发病年龄较低，预后分层多为中高危，TTT较短以及疾病侵袭性较强[3]–[4],[6],[11]。本研究11例患者中位发病年龄为55（51～72）岁，仅1例患者年龄大于65岁，发病年龄相对较低。根据CLL-IPI评分系统，中高危及极高危患者8例，而不伴t（14;19）（q32;q13）的CLL患者的危险度分层多为中低危[4],[6]。

有研究认为，仅存在t（14;19）（q32;q13）不足以诱导肿瘤的发生。Eµ-BCL3转基因小鼠模型提示，BCL3高表达可引起成熟B细胞在骨髓、淋巴结和腹腔中的增多，是B细胞肿瘤形成过程中的一个重要步骤，但其表达不足以引起B细胞恶性转化，需要获得额外的遗传畸变才能导致细胞发生恶性转化[12]。本研究中有3例患者仅存在t（14;19）（q32;q13），1例尚存在NOTCH1、KRAS等基因突变，另2例未行二代测序检测。文献报道的t（14;19）（q32;q13）阳性CLL病例中，15％～30％伴有复杂核型[4],[6]。而Baliakas等[9]报道的欧洲多中心研究显示，5 290例CLL患者中有271例患者出现复杂核型，约占5.1％，表明t（14;19）（q32;q13）阳性的CLL患者有更高比例的复杂核型。本研究中有6例患者为复杂核型，较文献报道的比例高，其中高复杂核型4例。复杂的细胞遗传学异常，尤其是高复杂核型，已成为与较差预后相关的新型独立生物学标志物之一[13]。+12是CLL中常见的染色体异常[14]，在我们报道的11例t（14;19）（q32;q13）阳性的CLL中，4例患者伴+12。除此之外，RB-1基因缺失、ATM基因缺失也是CLL常见的细胞遗传学异常，但在本研究中，11例患者均未见RB-1基因、ATM基因的缺失。

CLL患者外周血有典型的免疫表型：CD5（+）、CD19（+）、CD23（+）、CD43（+）、CD200（+）、CD10（−）、FMC7（−），弱表达sIg、CD20、CD22及CD79b，表达水平低于正常B细胞（dim）[2]。本研究11例患者中，3例患者免疫表型积分为3分，其中2例患者表达FMC7，未见CD79b表达减弱或缺失，1例患者不表达CD5，3例患者均未见CD22表达减弱或缺失。这3例患者中，2例为单纯性t（14;19）（q32;q13）。文献报道，具有BCL3易位的淋巴瘤多为CD20阳性B细胞淋巴瘤[3]，本研究中有8例患者流式细胞术提示CD20表达阳性，3例患者弱表达CD20。

伴IGH易位的CLL患者的基因突变谱仍然未知。有研究对46例IGH易位的CLL患者进行二代测序分析，发现NOTCH1、BCL2、FBXW7、ZMYM3和MGA的突变频率显著高于IGH易位阴性的CLL患者[15]。本研究中，7例患者进行了二代测序检测，突变频率较高的基因为NOTCH1（3/7）、FBXW7（3/7）和KMT2D（2/7）。所有患者均出现至少2个基因突变。虽然本研究例数不多，但为伴有该细胞遗传学异常的CLL患者的基因突变谱及预后分层提供了更多信息。

t（14;19）（q32;q13）可以导致位于19q13的BCL3基因与位于14q32的免疫球蛋白重链（IGH）基因座的调节元件并置，IGH基因的增强子导致BCL3高表达。BCL3编码 IKB 样蛋白并调节NF-κB转录因子的活性[12]。B细胞受体信号抑制剂（BTK抑制剂）在阻断NF-κB通路中发挥作用，因此也可能成为这一患者群体的替代疗法之一。

t（14;19）（q32;q13）还可以见于边缘区淋巴瘤、弥漫大B细胞淋巴瘤、套细胞淋巴瘤、侵袭性B细胞非霍奇金淋巴瘤等[1]。本实验室尚见两例t（14;19）（q32;q13）IGH::BCL3阳性的其他类型B细胞肿瘤，1例为弥漫大B细胞淋巴瘤，1例为免疫表型评分为2分的小B细胞淋巴瘤。

CLL患者中IGH易位并不多见，在CLL中占3％～20％[3]–[4],[6]。IGH最常见的伙伴基因是BCL2[16]，其次是BCL3，另外还可见MYC、BCL11A等基因。在B细胞恶性肿瘤中出现的染色体易位t（14;19）（q32;q13），IGH的伙伴基因还可见CEBPA、SPIB等，另外由于核型分析的分辨率比较低，t（14;19）（q32;q12），IGH::CCNE1也可能被误认为是IGH::BCL3[1]。本研究所在单位对于CLL患者的常规细胞遗传学检查包括加入CpG-ODN和IL-2培养72 h的核型分析及FISH检测，FISH检测的探针包括RB-1/LAM1、TP53/CEP17、CEP12、IGH::CCND1等。IGH::CCND1探针用于与套细胞淋巴瘤的鉴别诊断[2]，在没有IGH::CCND1融合的情况下，IGH信号模式的异常也可以提示涉及IGH基因的其他易位，如IGH::BCL2、IGH::BCL3等。既往的研究及本研究的结果表明，在诊断CLL时，FISH检测中使用IGH分离探针需要进一步重视。

综上，t（14;19）（q32;q13）可涉及IGH::BCL3重排，是CLL少见的重现性染色体异常，伴有+12及复杂核型较为常见，可能提示预后不良，其临床预后价值需要大型多中心队列研究进一步验证。
